# Conductive Thread-Based Textile Sensor for Continuous Perspiration Level Monitoring †

**DOI:** 10.3390/s18113775

**Published:** 2018-11-05

**Authors:** Ji Jia, Chengtian Xu, Shijia Pan, Stephen Xia, Peter Wei, Hae Young Noh, Pei Zhang, Xiaofan Jiang

**Affiliations:** 1 Electrical Engineering Department, Columbia University, New York, NY 10027, USA; jj2860@columbia.edu (J.J.); cx2168@columbia.edu (C.X.); sx2194@columbia.edu (S.X.); pw2428@columbia.edu (P.W.); 2 Electrical and Computer Engineering Department, Carnegie Mellon University, Pittsburgh, PA 15213, USA; shijiapan@cmu.edu (S.P.); peizhang@cmu.edu (P.Z.); 3 Civil and Environmental Engineering Department, Carnegie Mellon University, Pittsburgh, PA 15213, USA; noh@cmu.edu

**Keywords:** textile sensor, sweat sensing, perspiration level monitoring

## Abstract

Individual perspiration level indicates a person’s physical status as well as their comfort level. Therefore, continuous perspiration level measurement enables people to monitor these conditions for applications including fitness assessment, athlete physical status monitoring, and patient/elderly care. Prior work on perspiration (sweat) sensing required the user either to be static or to wear the adhesive sensor directly on the skin, which limits users’ mobility and comfort. In this paper, we present a novel conductive thread-based textile sensor that measures an individual’s on-cloth sweat quantity. The sensor consists of three conductive threads. Each conductive thread is surrounded by a braided cotton cover. An additional braided cotton cover is placed outside the three conductive threads, holding them in a position that is stable for measurement. the sensor can be embedded at various locations on a person’s clothing. When the person sweats, the cotton braids absorb the sweat and change the conductivity (resistance) between conductive threads. We used a voltage dividing circuit to measure this resistance as the sensor output (DC). We then conducted a sensor calibration to map this measured voltage to the quantity of electrolyte solution (with the same density as sweat) applied to the sensor. We used this sensor to measure individuals’ perspiration quantity and infer their perceived perspiration levels. The system is able to limit the average prediction error to 0.4 levels when compared to five pre-defined perceived perspiration levels.

## 1. Introduction

Perspiration (sweating) is one of the mechanisms with which the human body regulates its thermal stability. The amount a person sweats can be used as a significant physiological marker to infer their health condition, comfort level, emotional state, and exercise level [1,2]. For example, in patient or elderly care applications, the perspiration level of patients or the elderly can be used to infer their comfort level, even if the subjects cannot express it on their own. Sensing perspiration conditions before subjects lose thermoregulation ability allows patients or medical professionals to identify discomfort in time and thus prevent problems such as hyperthermia or even death [2,3,4]. Furthermore, in non-medical situations, sweat level can serve as a physical reference for fitness assessment [5] and an indication of hyperhidrosis [6]. Therefore, monitoring an individual’s perspiration level can help obtain essential information on user comfort and health.

Prior works have explored approaches to detecting sweat conditions [7,8,9,10,11,12,13,14], which we discuss in detail in Section 2. General concerns that appear in these works are mainly three-fold: (1) the studies are performed on stationary subjects; (2) the sensors require special mounting to body or skin, which may cause discomfort and irritate sensitive skin; and (3) some of the sensor designs target only sweat component analysis and do not measure the quantity of sweat [12].

**Figure 1:**
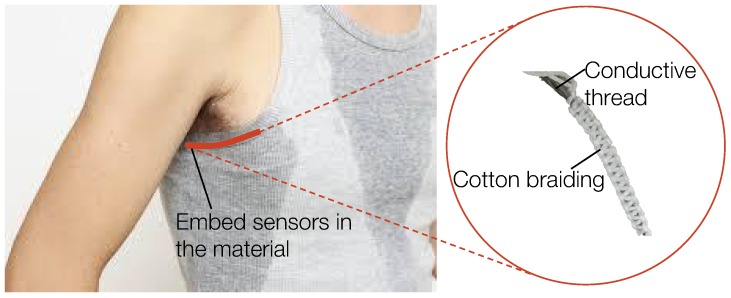
Sweat sensing through a conductive thread-based textile sensor with 3D structured cotton cover.

In this work, we target these aforementioned problems and present a new sensor design that measures an individual’s perspiration quantity. Our textile-based sensor is comfortable to wear, robust against body movements, and can be manufactured at large scale and low-cost. The purpose of the design is to measure the resistance between conductive threads, which changes according to the amount of sweat absorbed by the cotton braids between the threads [15]. However, weaving conductive threads directly into cloth does not guarantee stable and constant separation between cotton braids and conductive threads, especially when subjected to movement. To maintain a stable structure, we designed a second, structured cotton cover. Three conductive threads with cotton braids were then positioned to form a stable triangular structure and an external cotton cover was braided to keep them in position, as shown in Figure 1. The three-thread design allows not only stable sensor structure even during strenuous activities, but also multiple measurements to average, which ensures stable measurements. Besides usability advantages, our sensor is low-cost and production ready with standardized techniques for machined weaving [16]. The prototype sensors used for experiments in this paper are made by hand. If manufactured by commercial machinery, the consistency of the braiding would improve, increasing the stability of sensing performance.

In summary, here are the contributions of this work:

- We present a 3D structured, conductive, thread-based textile sensor that measures sweat quantity and is robust against user motion and sensor deformation.
- We calibrated the sensor by mapping the sensor output voltage and the on-sensor sweat quantity, and we conducted experiments under different sensing conditions, including motion and sensor deformation, to show sensing robustness.
- We present a perspiration level sensing system that utilizes the designed conductive-thread-based textile sensor to measure on-body sweat quantity and their perceived perspiration levels.

The rest of the paper is organized as follows: In Section 2, we review prior works on sweat sensing and compare differences between our sensor and the existing technologies. In Section 3, we introduce the overview of our sweat level monitoring system, followed by Section 4, where we describe in detail the design of our sensors. Then, we present the rest of the sensing system including sensor characterization (Section 5) and sweat level estimation based on readings (Section 6). We evaluate the system with both a sensing condition characterization experiment and a human experiment in Section 7. Finally, we discuss the applications and potential limitations in Section 8 and conclude the paper in Section 9.

## 2. Related Work and Background

We first introduce the background of the measurement method we present in this paper. Then, we discuss the related work and explain the research gaps our work fills.

### 2.1. Moisture Quantity Measurements

Studies have been done on moisture sensing through conductivity and resistance profiling. This method has been applied for both soil monitoring [17,18,19] and air humidity sensing [20,21]. The general idea is to measure the resistance changes between two points on a target that can hold/absorb a conductive solution (e.g., soil). We adopted a similar mechanism to one that has been used to measure moisture quantity in soil and air. To fit our purpose, we present a novel sensor design so that the sensor can be embedded into everyday clothes and measure the moisture (sweat) quantity of various body parts.

### 2.2. Sweat Conductivity

Human sweat is derived from blood plasma, and it contains electrolytes such as sodium which makes it conductive [22]. The conductivity of sweat has been studied for various purposes. It can be used as a diagnostic test for cystic fibrosis [23,24]. It has also been used as an indicator of sweat quantity [13]. These prior studies indicate that sweat quantity can be inferred through the conductivity of sweat absorbed by cotton threads, which is the foundation of our design.

### 2.3. Textile Sensors and Non-Intrusive Sensing

Textile sensors are designed to achieve non-intrusive sensing [25]. They have been used to achieve various sensing goals including but not limited to sweat pH levels [8], ECG, EEG, EMG, respiration, etc. [25]. Wearable monitoring devices are shown to offer a wide range of opportunities for non-intrusive long-term patients monitoring [26]. We design our textile sensor specifically for perspiration level monitoring and we take the non-intrusiveness as well as robust to motion and sensor deformation into account in our design philosophy.

### 2.4. Sweat Quantity Measurements

Various sensor designs have been explored to measure sweat quantity on the human body. Electrical impedance and thermal conductivity have both been adopted to measure the hydration of skin [7,9]. However, this pioneer research was done on users in a stationary condition [10], which may be impractical in a real-world scenario. Merilampi et al. proposed and demonstrated that it is possible to achieve binary measurements by scanning the frequency response of the passive UHF RFID textile tags [11]. They focused only on whether the target person sweats instead of measuring amounts that indicate the comfort level of the user. Wei et al. [10], Liu et al. [13], and Seno et al. [14] presented sensors that measure sweat rate and amounts through a sweat absorber and its capacitance [10] and conductivity [13,14], respectively. These sensors need to be directly mounted to people’s skin, which may lead to inaccurate readings on hairy skin and irritation on sensitive skin.

## 3. System Overview

**Figure 2:**
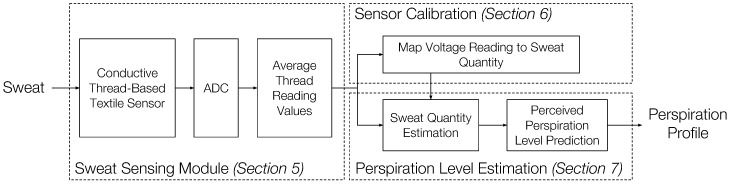
Perspiration level monitoring system overview.

Our perspiration level monitoring system consists of three parts, as shown in Figure 2: the sweat sensing module, the sensor calibration, and the perspiration level estimation. The sweat sensing module contains our conductive thread-based textile sweat sensor [15] and an ADC module to measure the sensor output from two conductive threads. The sensing module averages the ADC readings corresponding to the two conductive threads in the sensor as the final measurement. The details of the design consideration are further discussed in Section 4.

To map the measured sensor output (in voltage) to the quantity of moisture between threads (in the unit of mg), we conducted sensor calibration. We applied known volumes of salt solution onto the sensor and measured the voltage output of the sensor. Then, we conducted a fitting based on a simplified equation of solution resistance, which we explain in detail in Section 5.

The final goal of the system is to measure sweat quantity and estimate the perspiration level of the individual. This measurement is conducted in the perspiration level estimation module. Once we obtained the mapping between measured sensor output voltage and sensor solution quantity, we used it to conduct real-time sweat quantity measurement. To do so, we found the sweat quantity value corresponding to the sensor output voltage on the fitting curve from the sensor calibration. We further predicted the individual perceived perspiration level based on the kernel smooth regression from their historical measurements.

## 4. Sensor Design

The design of our conductive thread-based textile sensor is the key to this work, which we explain in this section. The design targets two challenges. (1) it is non-intrusive and easy to wear; and (2) it is robust to the motions of whoever wears it.

**Figure 3:**
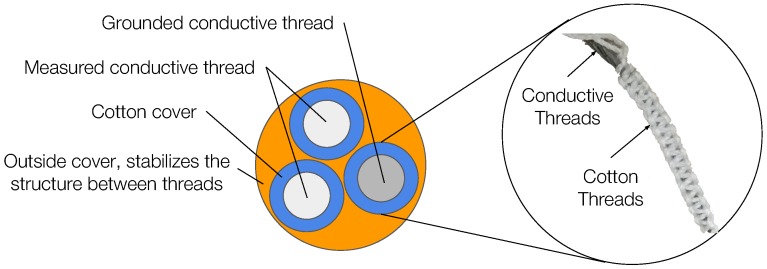
Cotton cover with square knots [15].

The sensor measures the conductivity of sweat to infer sweat quantity, therefore, an absorbent material is needed to “carry” the sweat during the measurement. Because cotton is one of the most absorbent materials and a common material for clothes, we decided to use it as an absorber for our sensor. To make the sensor non-intrusive, we decided to weave the absorber (cotton threads) and the conductive threads together to measure the sweat on the absorber (cotton threads). In this way, the sensor contacts the skin as part of the cloth without the need of being stuck to the skin directly. However, the traditional weaving that combines two types of threads cannot guarantee a stable connection between conductive threads when stretched or bent, which affects the measurements. To fix this issue and make the sensor robust to motion when people wear it, we designed the sensor with a 3D structure as follows. Each sensor contains three conductive threads covered by braided cotton covers. Then, we used an additional, external cotton cover to hold these three conductive threads together in a stable position for measurements. Figure 3 (left) shows a conceptual section view of our sensor, where the grey circles indicate the conductive threads, the blue circles represent the braided cotton cover outside the conductive thread, and the orange circle is the external cotton cover that holds these threads together. The stability of the blue circles and the relative position between conductive threads are crucial for the sensor design.

### 4.1. Conductive Threads Design

We place three threads in each sensor for the following reasons. From three conductive threads, we can obtain two pairwise voltage readings between the target thread and the grounded thread. We take the average of these two measurements as the final measurement. This enhances the sensor’s sensitivity for two reasons: (1) when the amount of sweat is low, e.g., only one side of the sensor directly contacts the sweat, averaging multiple threads allows the system to take multiple sweat–sensor contacting conditions into account and achieve a robust estimation of the sweat level; and (2) when human motion causes the thread to bend, which may affect the measurement, averaging multiple threads of different bending conditions can be more robust to the shape distortion-caused sensing variations. Another reason to have three conductive threads is that three threads will be packed together with a triangle sectional view, which is geometrically stable.

### 4.2. Cotton Cover Braiding

To sample the sweat, we placed a braided cotton cover on the surface of each conductive thread. We selected cotton because it is one of the most absorbent materials and is able to absorb up to 27× its weight in liquid [27]. Therefore, using cotton as absorber material provides a wider dynamic measuring range once applied to conductive threads. The braided cotton cover is built from cotton threads following the method of square knots, as shown in Figure 3. We choose square knots because it maintains a stable structure and is easy to implement.

Since the braided cotton cover serves as the absorber, the physical form of the cotton cover determines the sensors’ dynamic sensing range. In addition, the resistance of cotton fibers is determined by the whole range of moisture contents (i.e., 1–22.4%): LogR=−9.3logM+B, where *R* is the resistance of the sample in megohms, M the moisture content in percent of the dry weight, and B a constant. By rewriting the above equation, we get $R=B^{\prime }M^{-9.3},$ where $B=LogB^{\prime }$. This behavior of cotton traces back to the fact that a decrease in the moisture of cotton causes an increase in the general level of resistance [28].

### 4.3. Moisture Level to Voltage Conversion

To convert the moisture level between a pair of conductive threads into voltage output, we consider the cotton between them a resistor $R_{sweat}$, whose value varies with the moisture level (quantity of the sweat on the sensor). When the braided cotton cover is completely dry, its resistance can be considered infinite. On the other hand, when the braided cotton cover is saturated by sweat, its conductivity increases and the resistance decreases. To set up a reference level, we set the output to Vcc when the cotton is completely dry. Another known resistor $R_{ref}$ is then used to partake the voltage so that when the cotton is dry (where we consider $R_{sweat}=\infty$) the electric potential between $R_{sweat}$ and $R_{ref}$ equals Vcc.

**Figure 4:**
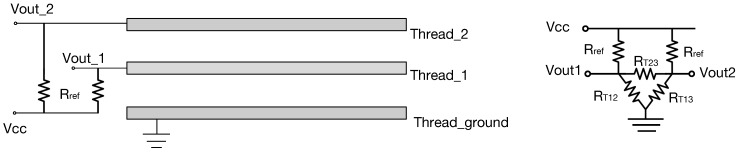
Sensor output circuit.

Figure 4 shows the circuit design. The output of the circuit is calculated through the equation below.

(1) $$Vout=Vcc\times R_{sweat}/\left(R_{sweat}+R_{ref}\right)$$

An equivalent circuit is shown on the right hand side of Figure 4, where $R_{T12}$, $R_{T13}$, and $R_{T23}$ are equivalent resistors between thread pairs Thread_1 and Thread_ground, Thread_2 and Thread_ground, and Thread_1 and Thread_2, respectively. We consider $R_{T12}$ and $R_{T13}$ here as the $R_{sweat}$ in Equation (1). Ideally, when the solution is distributed onto threads evenly, the values of $R_{T12}$ and $R_{T13}$ are the same. It follows that the potential difference of $V_{out1}$ and $V_{out2}$ are the same and the resistance between Thread_1 and Thread_2 can be ignored.

## 5. Sensor Calibration

To enable sweat quantity measurement with our sensor in real-time, we need to find the mapping between the sensor output and the on-sensor sweat quantity. To accomplish this, we conducted sensor calibration with the series of experiments discussed in this section.

### 5.1. Sensor Calibration Experiment Settings

**Figure 5:**
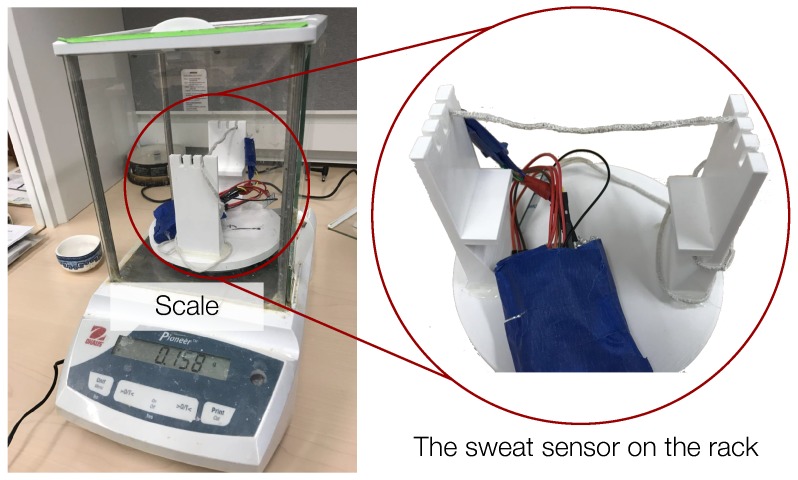
Sensor calibration experiment settings.

We conducted the calibration experiment in a room with consistent temperature and humidity. We used sodium chloride solution (salt water) as a sweat substitute for sensor calibration. Prior research shows that the sodium concentration of human sweat can vary between 117 and 172 mEq per L with an average of 137.8 mEq per L [29], which is approximately 8.06 mg per L for NaCl. Therefore, in the sensor calibration experiment, we used 1% sodium chloride solution as a substitute for human sweat [30]. Figure 5 shows the sensor calibration hardware setting. The tested sensor is fixed on a 3D-printed rack, and the entire rack is placed on a scale (the scale we used for measurements in this work is Ohaus Pioneer) that can measure weight differences up to 1 mg.

We built a 35 cm long conductive thread-based textile, and conducted multiple trials of calibration on different part of the textile, 10 cm each trial. Since the textile is weaved in one batch, we considered the behavior of different parts of the textile consistent. For each trial, we first applied approximately 300 mg of solution on the thread so that the braided cotton covers were saturated. For the 10 cm long textile, when the solution evaporates to around 150 mg, we started to observe the sensor reading changes, which indicate the sensor’s range. We recorded both the voltage reading and the scale reading when there was 1 mg change on the scale reading. These measurements are then used to generating the fitting function, which is further discussed in Section 5.2.

### 5.2. Sensor Reading Mapping

When we applied different amounts of solution to our sensor, it changed the resistance between two conductive threads. This change in resistance leads to the change in the sensor output of the division circuit. We believe our sensor fits the simplified electrical conductivity model as follows. The resistance between the two conductive threads can be calculated as

(2) $$R_{x}=R\cdot d/S$$

where *R* stands for resistivity [31], *d* the distance between the conductive thread, and *S* the conductive thread surface that is soaked by sweat, depending on the amount absorbed.

**Figure 6:**
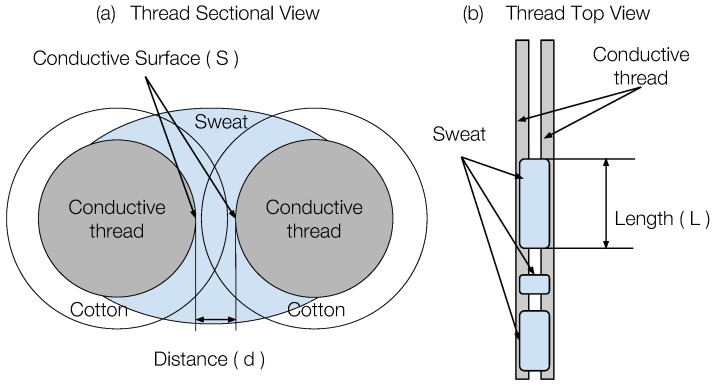
Simplified electrical conductivity model for our sensor [15].

Figure 6 illustrates this simplified model with sectional and top views of a pair of conductive threads. We used the minimum distance *d* between two conductive thread surfaces as a representative of the between-surface distance. Since the braided cotton cover maintains a stable structure even when in motion, we considered *d* a constant. When the sweat soaks on only part of the sensor threads, as shown in Figure 6 (right), the conductive surface on threads *S* is proportional to the length *L* of the soaking area. When more sweat is absorbed by the cotton cover, the effective conductive surface on threads *S* is larger. Therefore, based on the simplified model in Equation (2), the resistance $R_{x}\propto 1/q$, where *q* is the sweat quantity. As a result, we calibrate the sensor output and the measured on-sensor sweat quantity with the fitting function below.

(3) $$y=p_{1}/\left(x+p_{2}\right)$$

**Figure 7:**
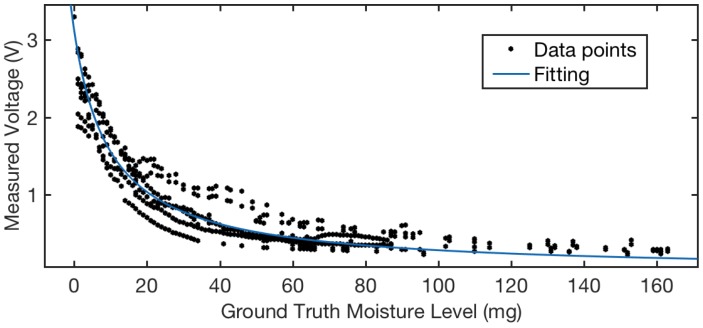
Sensor characterization: thread resistance vs. solution quantity [15].

We collected eight trials of calibration data, which are plotted in Figure 7. The calibration trials were conducted on 10-cm sensor sections in a room where the humidity and temperature are controlled and constant. Based on the calibration data plotted in Figure 7 as black dots, we observed that, when the solution quantity is less than 80 mg, the threads show a decreasing trend in measured voltage consistent with the increasing solution quantity. Most of the resistance changes correspond to the solution quantity change from 0 to 30 mg, where the difference in voltage is from 3.3 to 0.77 V. When the solution quantity changes from 30 to 80 mg, the variation between different trials is higher that that of other solution quantity ranges.

### 5.3. Dynamic Sensing Range and Sensitivity

Based on the calibration results, we further summarize the sensor’s dynamic sensing range and sensitivity here. When the solution quantity is beyond 80 mg, the voltage readings fluctuate at 0.35 volt, which means the braided cotton cover is saturated. Therefore, we consider the dynamic sensing range of the sensor is up to 80 mg. Since the calibration mapping is not linear, we analyze the sensitivity of the sensor at different sensing ranges based on the fitting curve obtained in Figure 7. Table 1 shows the target sensing range in the unit of mg, which corresponds to the x-axis in Figure 7. For each sensing range listed, we selected the median value of that range, and obtained the derivative of the fitting curve at that point. We considered this derivative value the sensitivity of the target range.

## 6. Perspiration Level Estimation

The system estimates two types of perspiration level information: the measured sweat quantity (Section 6.1) of the shirt and the perceived perspiration levels (Section 6.2). The measured sweat quantity values are absolute values in the unit of mg mapped to sensor readings. However, when used in real-life applications, it may be difficult for people to understand these absolute values of sweat quantity. For example, telling a person they sweated 100 mg does not help them know what that means in the context of their physical health. To help people connect sweat quantity values to an understanding of their physical and emotional health, we defined perceived perspiration levels by describing the expected physical conditions around different sweat quantities. The system estimates these individual perceived perspiration levels based on each person’s historical label data so that it is tailored to their own physical experience.

### 6.1. Sweat Quantity Estimation

**Figure 8:**
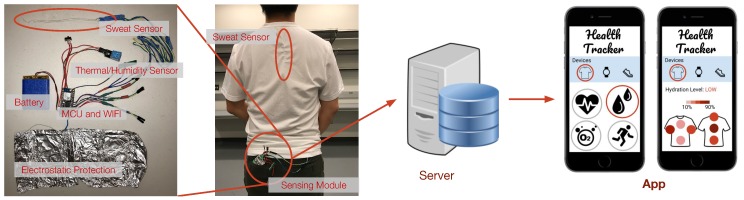
Perspiration level estimation. The sensor measures the sweat quantity of the cotton threads. The system maps the measured voltage to the sweat quantity in mg and estimates each user’s perceived perspiration level based on personal historical data. The measured sweat quantity and estimated perceived perspiration level are displayed on the mobile app [15].

The sensors measure the output voltage from sensors embedded at designated areas on the body and use the calibration fitting curve to calculate sweat quantity. Once the sensing module measures sensor resistance change in the form of divided voltage (DC), it sends the measurements to the server as shown in Figure 8. For each sweat sensor, the system obtains two output voltage values from the non-grounded conductive thread and calculates the average $V_{out\_avg}$. Then, the system maps the average voltage values to sweat quantity based on the mapping relationship learned from the sensor calibration, as discussed in Section 5.

### 6.2. Perceived Perspiration Level Prediction

The server calculates the sweat quantity in mg and estimates each user’s perceived perspiration level based on their historical data. To build the individual perspiration profile, we collected multiple trials of training data when the person exercised while wearing our sensors. Then, we applied kernel smooth regression between the measured sweat quantity and the recorded perceived perspiration levels. When an unknown trial is collected, the system predicts the perceived perspiration level based on the kernel smooth regression model built from the training data trials. Note that this is a personalized measurement. Therefore, individuals need to provide labeling to obtain their perspiration profile.

## 7. Evaluation

To evaluate our perspiration level estimation system, we build two sensors from the calibrated conductive thread-based textile, with one 10 cm textile each (Section 7.1). To demonstrate the robustness of the sensor under different sensing conditions, we conduct a series of experiments with controlled sensor motion/deformation (Section 7.2). Finally, we evaluate the system through a real-world human experiment with multiple participants exercising at different intensities (Section 7.3).

### 7.1. Implementation

We implemented the sensor design introduced in Section 4. In addition to previously described design challenges, we also faced several implementation challenges. First, since the sensor must be worn on the body during the experiments, resulting static charge may damage the board. To prevent this, we used a conductive foil to shield against electric fields induced by triboelectric charging and keep the electronics board isolated from the conductive foil, as shown in Figure 5.

In addition, since the working principle of the sensor is to measure changing resistance, if a person’s clothing contains any ambient conductive materials in a similar resistance range as the sensor, this could affect the sensing accuracy significantly. The resistance of the sensor changes from the magnitudes of 100k Ohm to over 100M Ohm. Therefore, ambient materials such as hot glue may affect the readings significantly. To prevent this, we protect the sensor with a high resistance cover to keep it appropriately isolated. The reference resistance $R_{ref}$ we used is 1 M Ohm.

Furthermore, to ensure the same cotton density at different parts of the sensor, the tension on the braiding needs to be the same. Braiding by hand makes it difficult to ensure even and consistent tension between cotton threads. For the first prototype sensor, we spent time keeping the force on the cotton thread as even as possible during the braiding. This can be easily controlled if manufactured in large amount through weaving machines.

### 7.2. Sensing Condition Characterization

To evaluate the sensor performance under real-world sensing conditions, we conducted a series of controlled experiments targeting: (1) sensor deformation; and (2) sweat concentration variation.

- Sensor Deformation. When people wear clothing with the embedded sensor, their motion may cause the sensor to continuously deform. Our sensor’s braided 3D structure makes the electrical condition between embedded conductive threads stable, keeping it robust to sensor deformation caused by human movement. In the following sections, we evaluate the robustness of the sensor under different motion velocity and bending angles.
- On-cloth Sweat Concentration. Different people have different sweat concentrations, as discussed in Section 5.1. When a user wears a sensor for a long duration, the sweat may accumulate on the sensor causing a high concentration of electrolytes. Therefore, it is important to understand the limitation of the sensing range regarding different solution concentrations.

#### 7.2.1. Condition Characterization Procedure

We conducted a series of experiments in which we applied the initial amount of solution to a sensor until the cotton on the sensor was saturated (the resistance value does not drop when more solution is applied). We measured and compared the sensor reading curves while the solution evaporated to demonstrate sensing consistency. To do that, we applied a 1% solution to the sensor until it was fully absorbed. Then, we measured the sensor readings while the solution was evaporating from the sensor under different motion conditions, at the sampling rate of 1 Hz. For trials investigating the same parameter, we kept environmental factors such as room temperature and humidity the same to keep the evaporation process consistent.

#### 7.2.2. Motion-induced Sensor Deformation

**Figure 9:**
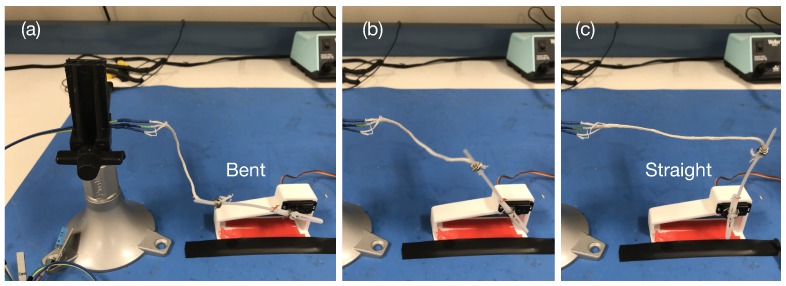
Sensor characterization with motion caused sensor bending. (**a**) fully bent, motor position is 90${}^{\circ }$. (**b**) half bent, motor position is 45${}^{\circ }$. (**c**) straight, motor position is 0${}^{\circ }$.

We evaluate the effect of motion-induced sensor deformation with the setup shown in Figure 9. To create consistent and comparable motion conditions, we fix one end of the sensor to a board holder as shown on the left hand of the setup, while fixing the other end of the sensor to a plastic arm connected to a servo motor. The servo motor is programmed to rotate between 0${}^{\circ }$ (Figure 9a) and 90${}^{\circ }$ (Figure 9c), which corresponds to the sensor positions bent and straight. We consider the motion from Position (a) to Position (c) then back to Position (a) as a complete rotation. The three motion conditions we investigated are: (1) completely static; (2) slow motion, i.e., 10 s per rotation; and (3) fast motion, i.e., 2.5 s per rotation.

**Figure 10:**
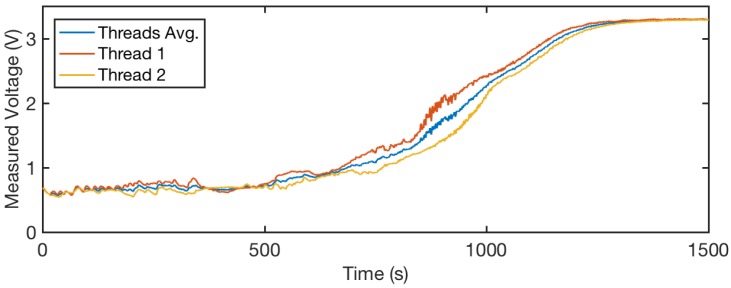
Thread readings during the evaporation process. We show the comparison between individual threads and their average value. The average shows a more stable reading than the single threads. This disparity may be caused by human motion and sensor bending.

We show sensor readings from Thread 1 and Thread 2 in slow motion in Figure 10. The red and yellow lines are sensor output values from Thread 1 and Thread 2, respectively. The blue line shows average readings of these two threads. We observe noise on Thread 1 between 900 and 1000 s, and there is relatively low noise on Thread 2 during that period. Therefore, by averaging the values from the two readings, we observe a stable curve that is robust to motion.

**Figure 11:**
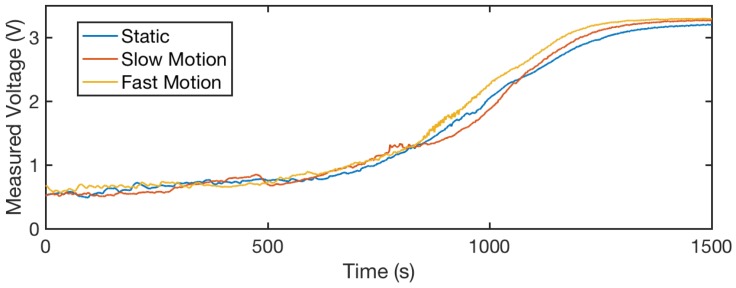
Thread readings during the evaporation process. We show the comparison between different levels of motion applied to the sensor, as discussed in Figure 9. We observe that under static, slow and fast motion, the sensor shows similar profiling in the evaporation process. This indicates that our sensor is robust to motion-caused distortion.

We further compare the evaporation processes of the three motion conditions in Figure 11. The sensor output values reported in this figure are the average readings from two threads. We observe that, despite the different motion conditions, the system shows consistent curves through the evaporation process. This consistency indicates that our sensor is robust to different levels of motion during the sensing process.

#### 7.2.3. Sharp Bending at Different Angles

**Figure 12:**
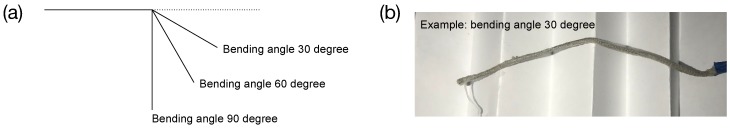
Sensor characterization with sharp static sensor bending: (**a**) the bending angle and its corresponding sensor position; and (**b**) a photo of the sensor with a bending angle of 30${}^{\circ }$ during the experiment.

Since the sensor may be bent at a constant sharp angle when embedded in clothes, we further evaluated the effects of sharp bending-caused sensor deformation on the sensor outputs. Sharp bending may cause a significant change in the distance between the conductive threads and the pressure on the cotton threads, causing a significant variation in sensor output. To characterize partial sharp bending and its effects on sensor outputs, we conducted a set of characterization experiments where the sensors were bent intentionally to a specific degree, including 30${}^{\circ }$, 60${}^{\circ }$, and 90${}^{\circ }$. Figure 12 demonstrates: (a) the different bending angles of the sensor; and (b) how we kept the deformation angle during the experiment.

**Figure 13:**
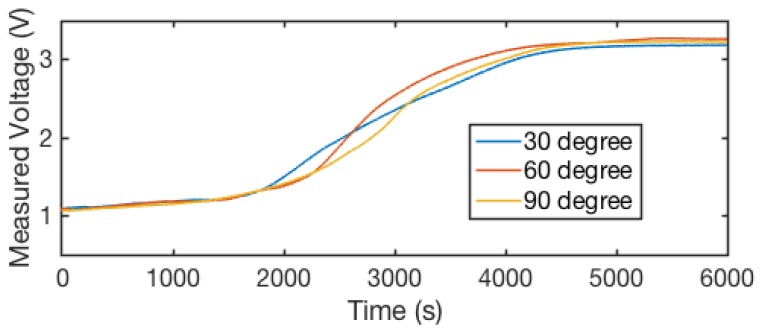
Thread readings during the evaporation process, with a five-point moving average applied. We observed that, under different bending angles, the sensor shows similar profiling through the evaporation process. This indicates that our sensor is robust to bending distortion.

Figure 13 shows the sensor output values when the sensor is bent at different angles during the evaporation progress. The sensor output values reported in this figure are the average readings from two threads. We observed that the sensor output curves for different bending degrees are consistent. These consistent measurements indicate that the bending angle investigated does not affect the sensor reading, and our braided 3D structure is robust to deformation from angles up to 90${}^{\circ }$.

#### 7.2.4. Variation in Solution Concentration

When different people have different sweat concentrations or their accumulative sweating changes the sweat concentration on the sensor, the high concentration of electrolytes may alter the sensibility of the sensor. To investigate the effects of different sweat concentration levels, we conducted our characterization experiment with four different levels of solution: tap water (we considered the measurement with tap water as the zero condition) as well as 1%, 2%, and 4% salt in tap water. Note that after each experiment the sensor was washed to ensure no residual electrolytes remain on the sensor.

**Figure 14:**
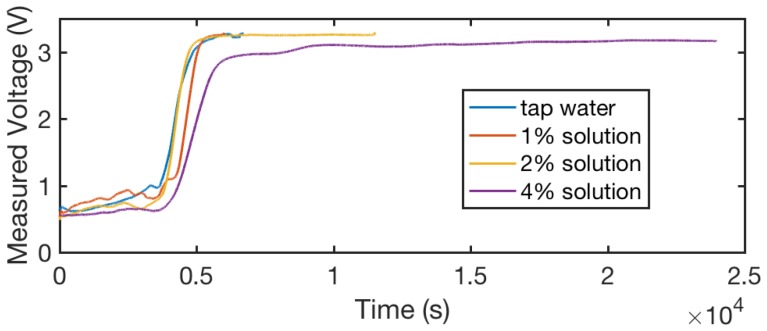
Thread readings during the evaporation process, with a five-point moving average applied. We observed that, for different solution densities below 4%, the sensor shows similar profiling through the evaporation process, while, when the solution density is as high as 4%, the voltage increase rate (resistance decrease rate) slows down. This could be caused by the combination of a high density of electrolytes and a high humidity environment.

Figure 14 shows the curves of sensor outputs—average voltages from two threads—under these four concentration levels during the evaporation process. As discussed before, the 1% solution is a similar electrolyte concentration to human sweat [30]. We observed that for the solution that is significantly higher than sweat—the 4% solution—the measured voltage does not return to 3.3 volt after it dries, which may be caused by the high concentration of the electrolytes, which are conductive when the humidity level in the air is high. The low concentration solution we investigated demonstrated a consistent performance across the evaporation process. Our experiment indicates that the sensor’s sensitivity and dynamic range remains consistent for most people’s sweat concentration range (the variation is less than 2× the average concentration) [29,30].

### 7.3. On-body Experiments

To evaluate our sweat sensing system under a realistic scenario, we further experimented with human subjects while embedding the sweat sensors at different locations on a polyester shirt. In this section, we discuss how sweat sensing on different body parts at different activity levels vary among individuals.

**Figure 15:**
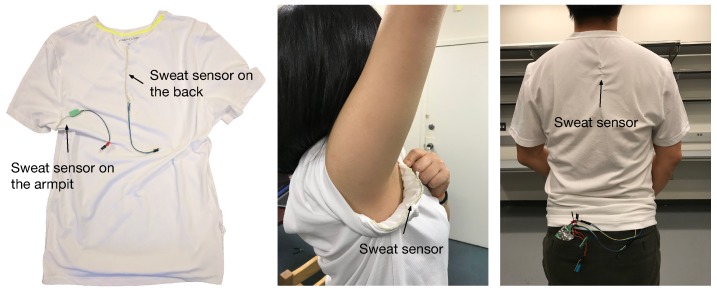
On-body experiment settings and sensor calibration. Two sweat sensors were sewn into a shirt at similar locations to conduct the sensor calibration before beginning the human experiments.

As discussed in Section 6, the system outputs two types of sweat level information: the measured sweat quantity and the perceived perspiration levels. We conducted multiple trials of exercise experiments on one participant and used their historical perceived perspiration level to build a prediction model. We applied the prediction model to the testing data and calculated the prediction error to evaluate the system performance. To investigate the sweating behavior of a human subject, we sewed two sensors (10 cm each) into the back and armpit of a shirt, as shown in Figure 15. The armpit and back are two of the sweatiest parts of the body because there are a relatively high density of sweat glands there.

#### 7.3.1. Sweat Quantity Estimation Evaluation

**Figure 16:**
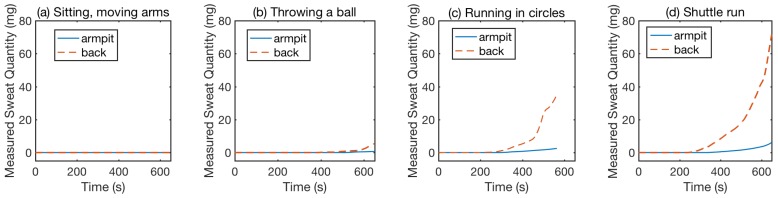
Measured sweat quantities from sensors at different locations when a person conducts different activities [15].

To demonstrate the performance and robustness of our system, we conducted experiments in which the participants performed activities with different sweating profiles, including moving arms while sitting, throwing a ball against the wall, running in a circle and shuttle runs. The sensing results are shown in Figure 16. Blue solid lines are the sensing values from the sensor sewn into the armpit and red dash lines are the sensing values from the sensor sewn into the back. We analyzed the system performance based on Figure 16 from two aspects: the activity comparison and the body parts comparison.

##### Activity Comparison

We selected four activities as representatives of four different activity levels: (1) moving arms; (2) throwing a ball; (3) running in a circle; and (4) shuttle run. We used our system to detect sweating profiles on the human subject while they performed these activities. The arm moving is the mildest activity. Therefore, after 650 s, readings from both sensors are still around 0 mg, as shown in Figure 16a. When the participant throws a ball, the activity level is higher, which causes the back and armpit sensor readings to increase after 500 s of activity, as shown in Figure 16b. For more intense activity (e.g., running), the sensor reading starts to increase even earlier—around 300 s after the participant begins the activity—as shown in Figure 16c,d. However, for the two different types of running, even though the participant experiences different levels of activity intensity, the measured sweat sensor readings show similar change trends and rates. Based on information from the personal trainer we consulted, the duration of the exercise affects the sweat rate and quantity more than intensity, which explains why the intensity of the exercise does not correlate to the measured sweat quantity.

##### Body Part Comparison

We placed two sensors at two locations that are sweat-heavy for most people: (1) armpit; and (2) back. We observed that, for the same participant conducting different activities, the back always sweats more than the armpits. For the moving arm activity, no body parts recorded clear sweat, as shown in Figure 16a, due to the non-intensity of the activity. For the throwing a ball activity, the measured sweat quantities are shown in Figure 16b, the back sensor detects more sweat than that on the armpit after 500 s. Similarly, for the rest of the activities that are more intense than throwing a ball, as shown in Figure 16c,d, the sensor on the back shows a faster increasing trend than that of the armpit.

#### 7.3.2. Perceived Perspiration Level Prediction Evaluation

Individuals perceive their perspiration levels differently based on their physical sensations. To further evaluate the system’s ability to estimate the perceived perspiration levels of people based on measured sweat quantity, we considered five different levels of perceived perspiration, as summarized in Table 2.

We selected running in a circle as the activity to test, and each participant ran multiple trials in a room with a stable temperature. During the experiment, we encouraged the participants to keep themselves hydrated. We also asked participants to answer the question: “How wet do you think your shirt is? Using a scale from 1 to 5, where 1 is dry, and 5 is soaking wet.” Below, we first analyze the perceived perspiration level profile from three different participants and then discuss how to predict individual perceived perspiration levels based on personal historical sweat sensing data.

##### Individual Perceived Perspiration Level Comparison

**Figure 17:**
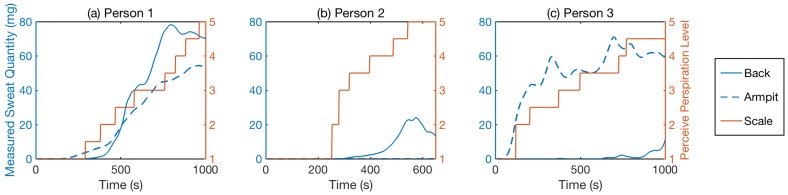
Perceived perspiration level based on people’s lived experience ((**a**–**c**) represent three different people). The x-axis is the time from the moment they start to exercise until they stop. The solid blue lines are the measured sweat quantity (mg) for the sensor on the back. Blue dash lines are the measured sweat quantity (ms) for the sensor at the armpit. The red line is their perceived perspiration level, as listed in Table 2 [15].

Figure 17 shows examples of three participants marking their perceived perspiration levels (as listed in Table 2) and the corresponding sensor measurements. We observed that the sensor readings between the “start to sweat” and the “heavy sweat” level cover the most sensitive range. This perceived sensing range corresponds to the measured sweat quantity below 60 mg in general, which is the sensitivity of the sensor, as discussed in Table 1.

In addition, different people may have different sweat profiles. For example, Person 1 and Person 3 have heavy armpit sweat, while Person 2 sweats on the back faster and has a lower tolerance to sweat on their shirt. The perceived perspiration levels of Person 1 and Person 3 have a stronger correlation to the measured sweat quantity at the armpits than that of Person 2. Person 3 gets sweaty quickly, and her armpit sweat shows a significant increase in the first 100 s. However, her back sweat profile only goes up to around 15 mg during the entire exercise. This is because the sports bra that the participant wore absorbed most of the sweat and made the absorption of the sweat on the sensor more difficult and uneven. For female users, we can address this by embedding the sensor on the edge of the bra.

##### Perceived Perspiration Level Prediction

**Figure 18:**
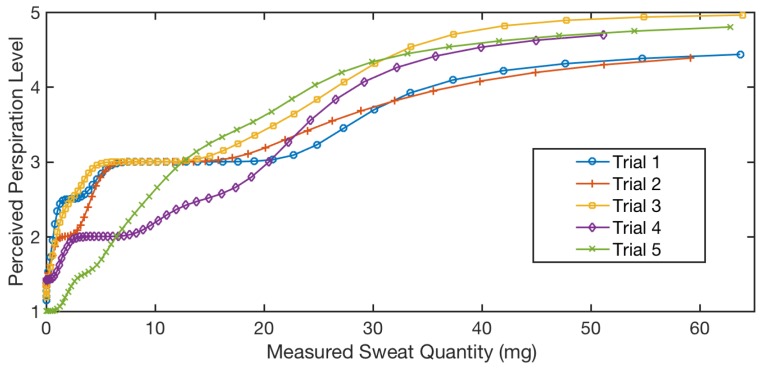
Perceived perspiration level base on people feeling vs. the measured.

We conducted multiple exercise trials on the same person and collected the measured sweat quantity together with their perceived perspiration level. For each trial, we applied kernel smooth regression between the measured sweat quantity and the recorded perceived perspiration level. Figure 18 shows the regression of five different trials from one participant.

Even though the trials show different characteristics, the overall trends are similar, and the perceived perspiration level difference for the same measured sweat quantity is mainly within one level. Therefore, we further utilized one trial as the testing data and the rest as the training data to conduct predictions using the kernel smooth regression model built upon the training data. When we conducted cross-validation, the average perceived perspiration level estimation error was 0.4 with a standard deviation of 0.28.

## 8. Discussion

In this section, we further discuss the potential extension of this work. First, we focus on cotton thread structure and how it may affect the sensor sensibility. Then, we discuss how this technology can be applied in a large-scale deployment to obtain environmental information from individual physical responses.

### 8.1. Sensitivity and Sensor Structure

We utilized the 3D braiding structure introduced in Section 4.2 to achieve stable sensing of sweat quantity levels. In our sensor characterization experiment, we observed that, as it absorbs more sweat, the sensitivity of the sensor decreases. We measured the dynamic sensing range of the sensor in this implementation, which was 0 to 80 mg. The 3D braiding structure, as well as its material, can both affect the sensitivity and the dynamic sensing range of the sensor.

Cotton thread thickness is one of the potential structural parameters that affects the sensitivity of the sensor. If the cotton thread used for braiding is thicker than the thread used in this implementation, the distances between conductive threads will increase, which will result in higher resistance under the same amount of sweat. As a result, the sensor will have less sensibility to low moisture levels but higher sensibility to high moisture levels. In this case, the sensitivity of the sensor will decrease, because the V/mg that can be measured decreases with the increase of the dynamic sensing range. If the sensor length increases, the sensitivity of the sensor does not change. However, when used in a real-world scenario, a longer sensor has a larger sensing surface, covers a larger skin surface and obtains more sweat, leading to a higher sensitivity when the sweat level is low. We selected cotton thread to braid the 3D structure as a sweat absorber. We plan to also explore other materials that affect the sensitivity of the sensor. For example, if a material such as nylon is used, the evaporation speed of the sweat is higher. Therefore, when using a different material, the evaporation rate can be used to calibrate the sensitivity model as well as the dynamic range. We focused on the feasibility of the sensor in this study, and we plan to conduct more sensor structure and material related studies with machine woven sensors for manufacturing consistency as well as the practicality.

### 8.2. Sweat Level Crowd-Sourcing for Environmental Condition Inference

We have discussed above potential applications such as monitoring a subject’s hyperthermia condition or exercise levels. We have also explained that the sensor could be used to predict an individual’s perceived perspiration level based on the measured sweat quantity. We foresee that, when the sensor is applied in a large-scale deployment (such as on patrons in an exercise gym or members of a sports team), the crowd-sourced sensing data can be further used to infer the group condition and related information, such as if a specific area is too warm or cold based on sensed human thermal regulation.

## 9. Conclusions

In this paper, we present our conductive thread-based textile sensor that can be integrated into clothing for continuous perspiration sensing. The sensor consists of three cotton-covered conductive threads and measures moisture between the conductive threads to infer sweat quantity. Each cotton-covered conductive thread is braided into a protective 3D structure, which allows robust sweat quantity measurement despite the sensor deformation caused by human motions. We performed sensor characterization to discover the mapping between measured sweat quantity and sensor voltage output. To evaluate the sensor and the system, we conducted sensing condition characterization to demonstrate its robustness against sensor distortion due to motion, bending, and sweat variations (solution concentration). Experiments with the prototype were conducted with multiple volunteers to explore people’s sweat profiles. We further investigated predicting people’s perceived perspiration levels based on the sensing data and achieved an average error of 0.4 levels when applied to a five-level scale of human perception.

## 10. Patents

The sensor introduced in this work has been filed a patent in the US.

## Figures and Tables

**Table 1:** Sensor sensitivity.

Target Range (mg)	0–1	1–10	10–20	20–40	40–80
Sensitivity (V/mg)	0.2856	0.1392	0.0781	0.0194	0.0063

**Table 2:** Perceived Perspiration Level [15].

Scale	1	2	3	4	5
Description	dry	start to sweat	light sweat	heavy sweat	soaking wet
